# Continuing Medical Education via Telemedicine and Sustainable Improvements to Health

**DOI:** 10.1155/2016/2424709

**Published:** 2016-08-31

**Authors:** Fuhmei Wang

**Affiliations:** Department of Economics and Department of Public Health, Research Center for Energy Technology and Strategy, National Cheng Kung University, Tainan 701, Taiwan

## Abstract

*Background.* This research aims to investigate the quantitative relationship between telemedicine and online continuing medical education (CME) and to find the optimal CME lectures to be delivered via telemedicine to improve the population's health status.* Objective.* This study examines the following: (1) What factors foster learning processes in CME via telemedicine? (2) What is the possible role of online CME in health improvement? And (3) How optimal learning processes can be integrated with various health services?* Methods.* By applying telemedicine experiences in Taiwan over the period 1995–2004, this study uses panel data and the method of ordinary least squares to embed an adequate set of phenomena affecting the provision of online CME lectures versus health status.* Results.* Analytical results find that a nonlinear online CME-health nexus exists. Increases in the provision of online CME lectures are associated with health improvements. However, after the optimum has been reached, greater provision of online CME lectures may be associated with decreasing population health.* Conclusion.* Health attainment could be partially viewed as being determined by the achievement of the appropriately providing online CME lectures. This study has evaluated the population's health outcomes and responded to the currently inadequate provision of online CME lectures via telemedicine.

## 1. Introduction

Online continuing medical education (CME) for physicians can be carried out via telemedicine systems. Telemedicine systems thus offer new ways to practice medicine and enable the remote delivery of personal health services, continuing medical education, and patient health education [1].

An extensive international literature has reported on the efficacy of telemedicine [2, 3] and clinical outcomes of telemedicine [4–6]. Little attention has been paid to the benefit reported for practitioners [7, 8], despite this being a rationale for the use of telemedicine in rural and remote areas [1]. Physicians are reported to benefit from telemedicine by increased contact with specialists via the telecommunications system [6, 7]. The appropriate use of online CME via telemedicine has the potential to contribute to the improvement in the population's health through increased access upskilled health professionals and enhancing confidence of the rural health workforce.

However, the provision of more online CME might crowd out the time that a physician can devote to medicine practice and thus worsen the health status of his or her patients. The relation between online CME and health may thus be nonmonotonic, and there could be an optimum amount of online CME that can achieve sustainable improvements in health. Public health researchers use life expectancy at birth as a good proxy that reflects population's health [9], and this is also used in the current study to evaluate variations in health.

Our study contributes to and improve upon earlier studies in the following ways. First, our outcome measure of life expectancy on the effects of online CME via telemedicine permits us to estimate the optimal provision of telecommunications health professionals. Second, this study is among the first to integrate technology development and healthcare provision to highlight the effectiveness of online CME via telemedicine in sustainable health improvement. Third, the telemedicine system in Taiwan is a good model system that other countries can learn from when seeking to provide the accessibility and affordability of healthcare for rural residents as well as health professionals for remote physicians. This study thus aims to investigate what factors affect online CME provision in healthcare systems and what the optimal online CME lectures are for improving health.

## 2. Methods and Specifications

### 2.1. Factors Affecting Online CME Provided

The telemedicine program provides real time teleconferencing, transfers medical data for consultation, and increases confidentiality of health workers and patients in remote areas. The CME lectures that are provided through the educational technology system may be affected by the healthcare system as a whole, including factors such as telecommunications and face-to-face programs used, as well as medical resource concerns, including per capita gross domestic product (GDP) and per capita national health expenditure (NHE). The online CME that is provided could thus be a function of the following factors:

(1) Online CME=fTel. care, per capita GDP, per capita NHE, Con. services

in which Tel. care presents the quantity of telemedicine health services and Con. services represent conventional health services.

### 2.2. Online CME and Health

Physicians spend time on CME activities to cover the full range of topics important to their professional development. Research has shown that CME is an effective tool for changing physician practices and improving patient care [10–12]. Online CME thus aims to improve physician performance and the health status of their patients. There is a potential trade-off between online CME and other economic and healthcare factors that possibly affect health status. A rise in the lectures provided by online CME can affect health status through two channels. The first is the health crowding-out effect, whereby an increase in online CME lectures reduces activity with regard to other healthcare factors and the economy's resources which might also improve health status. This channel tends to deteriorate the population's health. The second is the effects of health improvement, whereby an increase in online CME lectures tends to improve the quality of healthcare. This channel leads to better health status. The net effect of a rise in online CME lectures on health status thus depends upon the relative strength of these two channels. The relationship between online CME lectures and health status is possibly nonlinear, and this needs to be examined. Healthcare provision through in-person and telecommunications systems has also unclear health effects, due to similar concerns. This research thus incorporates these ambiguous health effects and tries to find the optimal level of online CME lectures, as follows:

(2) Life exp.=ω1  online  CMEit+ω2  online  CMEit2+ω3Xit+εit,t=1,2,…,T;  i=1,2,…,N,

where Life exp. represents the population's life expectancy, online CME_*it*_ represents the number of online CME lectures, online CME_*it*_^2^ represents the square of the number of online CME lectures, *ε*_*it*_ represents an idiosyncratic error term, and *i* and *t* represent hospitals and time period, respectively. The vectors of *X*_*it*_ are composed of healthcare and economic explanatory variables.

### 2.3. Subjects

The subjects of this study are to examine the following: (1) What factors foster learning processes in the CME context in telemedicine? (2) What is the possible role of online CME in the context of health improvement? And (3) How optimal learning processes can be integrated with various health services? The results of this investigation can serve as paradigms for providing optimal online CME via telemedicine, thus improving the population's health.

### 2.4. Method

In econometrics, panel data can contain multidimensional data and observations on multiple phenomena observed over multiple time periods for the same hospitals or patients [13]. By applying telemedicine experiences in Taiwan over the period 1995–2004, this study uses panel data and the method of ordinary least squares for the multiple regression model with Stata 10.0 (Stata Corp., College Station, TX) and aims to embed an adequate set of phenomena affecting the provision of online CME lectures versus health status.

### 2.5. Data Description

In Taiwan, telemedicine services were first introduced in 1995 for physicians in remote sites in order to provide healthcare in rural areas. Such services included online CME for physicians and special medical services for the elderly, the handicapped, and terminally ill patients at home. The National Health Insurance (NHI) system was established in the same year, and enrollees enjoy almost free access to healthcare, with only a small copayment in most clinics and hospitals. However, in 2004, a telemedicine cost-benefit analysis conducted by the government cast doubt on the effectiveness of the system, and now its provision is limited to a number of qualitative pilot experiments.

Based on (1), the possible factors affecting online CME lectures are telemedicine health services (Tel. care), per capita gross domestic product in US dollars (per capita GDP), per capita national health expenditure in US dollars (per capita NHE), and conventional health services (Con. services).

Based on (2), we hypothesize that there is a nonmonotonic relationship between these variables and health status, which is represented by life expectancy (Life exp.). The focal variables include healthcare and economic factors. Healthcare factors include the number of online CME lectures (online CME), the square of the number of online CME lectures (online CME square), telemedicine expenditure in US dollars (Tel. Ex.), the square of telemedicine expenditure (Tel. Ex. square), conventional healthcare expenditure in US dollars (Con. Ex.), the square of conventional healthcare expenditure (Con. Ex. square), telemedicine health services (Tel. care), and conventional health services (Con. services). The economic factors are represented by per capita gross domestic product in US dollars (per capita GDP).

The panel datasets of dependent and explanatory variables are observed over the period 1995–2004 and are regarded as a national sample. This research is thus a retrospective study. The mentioned variables are collected from the Ministry of Health and Welfare and the Ministry of Interior, Taiwan.  Table 1 presents the summarized statistics.

## 3. Results

The panel data regression estimated results for the provision of online CME lectures and other selected variables are presented in Table 2. Increases in conventional health services (Con. services) and per capita national health expenditure (per capita NHE) are associated with increases in online CME lectures provided via telemedicine.

Per capita GDP negatively affects the number of online CME lectures provided via telemedicine. This is because with higher incomes the use of CME lectures via telemedicine may be substituted by a synthesis of different types of knowledge, which can be seen as a form of continuous medical training. Economic development is thus associated with decreases in the provision of online CME lectures. A 10% increase in the provided health services via telemedicine increases the provision of online CME lectures by 4.99%. The statistics for *R* ^2^ and the adjusted *R* ^2^ are 0.94 and 0.93, respectively, and these indicate that the selection of variables is valid and the overall fit of the specification is good.

Based on (1), apart from the provision of telemedicine services, the influences of Con. services, per capita NHE, and per capita GDP are averagely specified by incorporating the mean values from Table 1 into the constant term. The impact of telemedicine services on the provision of online CME lectures is estimated as

(3) $$\begin{array}{c} \text{Online CME lectures}=1306.8+0.499\text{ Tel. care}. \end{array}$$

**Figure 1:**
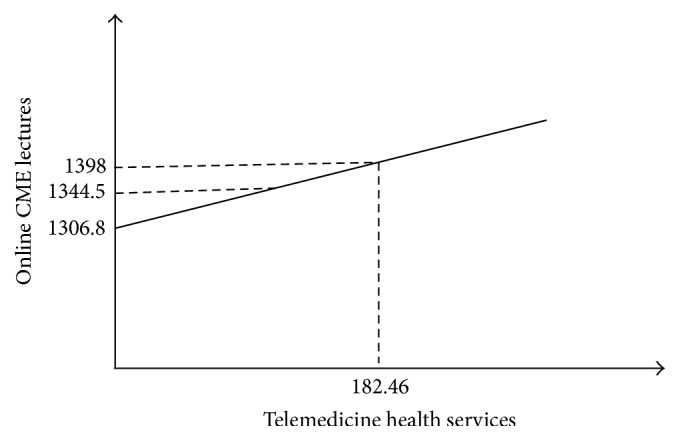
The relationship between telemedicine healthcare and the provision of online CME lectures.

Based on the statistics provided in Table 1 and (3), by substituting the real and estimated telemedicine services for online CME lectures, Figure 1 presents the positive relationship between telemedicine healthcare services and the provision of online CME lectures. This graph has an intercept on the vertical axis and shows that, even without telemedicine healthcare, the provision of online CME lectures through telecommunications should be at 1306.08 to meet physicians' needs. To reach the telemedicine health services at 182.46, which is the real level of provision, the number of online CME lectures should be increased to 1398 instead of the 1344.5 that is currently provided.

The main concern of this study is to explore the influences of healthcare factors on health status and to find out the optimum of the online CME lectures that should be provided. The multiple regression results for the application of health status to selected variables are presented in Table 3 and contain some interesting information. The statistics for *R* ^2^ and the adjusted *R* ^2^ are 0.99 and 0.99, respectively, and indicate that the selection of variables is valid and the overall fit of the specification is good. Online CME, conventional health expenditure and services, and per capita GDP all significantly affect health status. However, telemedicine expenditure, rather than the quantity of telemedicine services, affects health status. The explanation for this is that expenditure on the telemedicine system has the widespread effects, not only with regard to practicing medicine, but also for providing health consultations, and this can also lead to health improvements. The quantity of telemedicine services provided might thus not present the whole picture of such healthcare provision, and this may affect the health status insignificantly. Nevertheless, the provision of in-person health services affects the health status negatively and significantly. Fortunately, the magnitude of such effect is trivial. In a nation with an NHI system funded by government, the provision of unnecessary services is wasteful and may not help the population's health. Per capita GDP affects the health status negatively, and a 10% increase in per capita GDP leads to a 0.002% decrease in life expectancy at birth. Higher incomes could lead to high pressure working and living conditions, and these are associated with worse health status, which implies the possibility of a health fast lane effect, with higher incomes being associated with worse health [14].

We are interested in the nonlinear relationship between the provision of online CME lectures with health status. Based on (2), apart from the provided online CME lectures, the significantly influenced explanatory variables are averagely specified by the constant term, and the effects of provided online CME lectures on health status are estimated as

(4) Life exp. =74.84+0.0010182  online CME−2.56e−07online CME2.

**Figure 2:**
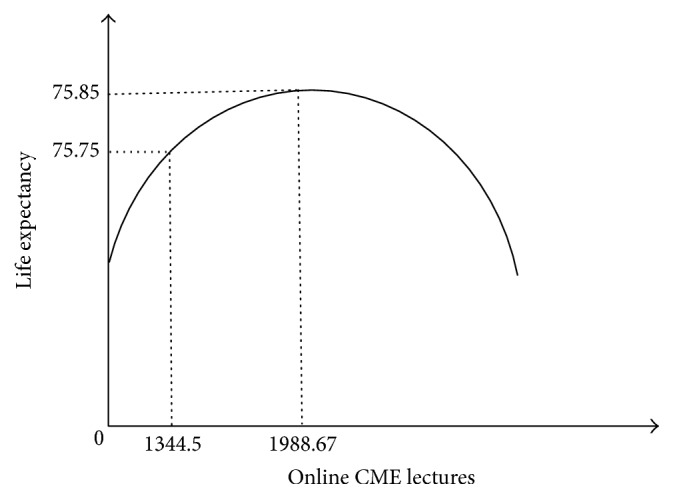
The relationship between online CME lectures provided via telemedicine and life expectancy.

Differentiating (4), we find that the optimal online CME lectures provided via telemedicine are 1988.67, with life expectancy at birth at 75.85. The real online CME lectures provided are less than this, being 1344.5, with life expectancy at birth at 75.75.  Figure 2 presents the inverse U-shaped relationship between the number of online CME lectures provided via telemedicine and health status.

## 4. Discussion and Conclusion

This research has discussed the effective ways of online and telemedicine system of medical services as well as presents rationale and need for online CME and provides evidence of online CME effects on health improvement. Subject investigated is current and relevant as governments decide to adopt and implement such mechanisms in technology and healthcare provision. Moreover, to our knowledge, the existing literature has not discussed the health sustainability of online CME via telemedicine.

A nonlinear online CME-health nexus exists. Increases in the provision of online CME lectures are not necessarily associated with better health status once the provision exceeds the optimal level. However, the results show that the real provision of online CME lectures in Taiwan is currently too low. The contributions of CME, a major facilitator of changes in practitioner behavior, are currently underprovided due to difficulties in CME delivery at remote sites. Moreover, the measures for health attainment could be partially viewed as being determined by the attainment of the appropriately providing online CME lectures. This research also finds that higher income could be associated with worse health, and health fast lane effects could explain fast rising health expenditures in developing and developed countries [14].

Telecommunications access is an increasingly important prerequisite to exploiting social, economic, and educational opportunities [15]. Evaluations of innovative technologies to support the provision of healthcare, such as telemedicine, have been centered on cost efficiency and the provider's perspective [16–18]. Measuring and improving the wellbeing of the population is a means of assessing the progress of a society and thus should be a focus of governments [19]. This study has evaluated the population's health outcomes in order to measure the effectiveness of physician performance and thus respond to the currently inadequate provision of online CME lectures via telemedicine.

## Figures and Tables

**Table 1:** Summary statistics.

Variables	Average	Std. deviation	Minimum	Maximum
Tel. care	182.46	155.2	25	645
Tel. exp.	166485	182218	9062.5	1.1 × 10^6^
Per capita GDP	13545	607.05	12769	14663
Per capita NHE	751.26	58.8	636.36	848.00
Online CME lectures	1344.5	1221.05	100	3066
Life exp.	75.75	0.47	74.85	76
Con. services	114 × 10^6^	607.05	100 × 10^6^	122.2 × 10^6^
Con. Ex.	15.7 × 10^9^	1356.5	13.3 × 10^9^	17.8 × 10^9^

**Table 2:** Regressions of online CME on selected variables.

Number of observations = 50						
Period of test = 1995–2004						
*F*(4, 45) = 164.72;						
Prob > *F* = 0.0000						
Root MSE = 301.91						
*R* ^2^ = 0.94;						
adjusted *R* ^2^ = 0.93						
Online CME	Coef.	Std. err.	*t*	*p* > |*t*|	95% conf. interval	

Tel. care	0.499	0.274	1.82	0.075^*∗∗∗*^	−0.053	1.052
Per capita GDP	−1.531	0.097	−15.731	0.000^*∗*^	−1.726	−1.335
Per capita NHE	22.395	1.320	16.97	0.000^*∗*^	19.736	25.053
Con. services	2.85 × 10^−5^	7.09 × 10^−6^	4.02	0.000^*∗*^	1.42 × 10^−5^	4.28 × 10^−5^
Constant	2520.839	1005.661	2.49	0.017^*∗∗*^	477.334	4528.344

^*∗∗∗*^Significant at 10%; ^*∗∗*^significant at 5%; ^*∗*^significant at 1%;

Online CME: continuing medical education via telemedicine; tel. care: the quantity of telemedicine health services; per capita GDP: per capita gross domestic product; per capita NHE: per capita national health expenditure; and con. services: conventional health services.

**Table 3:** Regressions of life expectancy at birth on selected variables.

Number of observations = 50						
Period of test = 1995–2004						
*F*(9, 40) = 36167.09						
Prob > *F* = 0.0000						
Root MSE = 0.00677						
*R* ^2^ = 0.99;						
adjusted *R* ^2^ = 0.99						
Life Exp.	Coef.	Std. err.	*t*	*p* > |*t*|	95% conf. interval	

Online CME	0.001	0.000	64.94	0.000^*∗*^	9.87 × 10^−4^	1.05 × 10^−3^
Online CME square	−2.56 × 10^−7^	4.13 × 10^−9^	−61.94	0.000^*∗*^	−2.64 × 10^−7^	−2.47 × 10^−7^
Tel. Ex.	15.40	0.092	167.79	0.000^*∗*^	15.214	15.585
Tel. Ex. square	−11.496	0.072	−160.74	0.000^*∗*^	−11.641	−11.352
Con. Ex.	−6.491 × 10^−4^	2.38 × 10^−5^	−27.25	0.000^*∗*^	−6.973 × 10^−4^	−6.01 × 10^−4^
Con. Ex. square	3.41 × 10^−8^	8.28 × 10^−10^	41.14	0.000^*∗*^	3.24 × 10^−8^	3.57 × 10^−8^
Tel. care	−8.55 × 10^−6^	6.47 × 10^−6^	−1.32	0.194	−2.16 × 10^−4^	4.52 × 10^−6^
Con. services	−3.43 × 10^−8^	4.23 × 10^−10^	−81.20	0.000^*∗*^	−3.52 × 10^−8^	−3.35 × 10^−8^
Per capita GDP	−2.289 × 10^−4^	5.04 × 10^−6^	−45.43	0.000^*∗*^	−2.39 × 10^−4^	−2.19 × 10^−4^
Constant	78.751	0.201	391.93	0.000^*∗*^	78.344	79.157

^*∗*^Significant at 1%;

Life exp.: life expectancy at birth; online CME: continuing medical education via telemedicine; online CME square: the square of online CME; Tel. Ex.: the expenditure on telemedicine in millions of US dollars; Tel. Ex. square: the square of Tel. Ex.; Con. services: conventional health services; Con. Ex.: conventional health expenditure in millions of US dollars; Con. Ex. square: the square of Con. Ex.; tel. care: the quantity of telemedicine health services; and per capita GDP: per capita gross domestic product.
